# Hydrophobic Components in Light-Yellow Pulp Sweet Potato (*Ipomoea batatas* (L.) Lam.) Tubers Suppress LPS-Induced Inflammatory Responses in RAW264.7 Cells via Activation of the Nrf2 Pathway

**DOI:** 10.3390/nu16040563

**Published:** 2024-02-18

**Authors:** Yuma Matsumoto, Mari Suto, Io Umebara, Hirofumi Masutomi, Katsuyuki Ishihara

**Affiliations:** Research and Development Division, Calbee, Inc., 23-6 Kiyohara-Kogyodanchi, Utsunomiya 321-3231, Japan; y_matsumoto1@calbee.co.jp (Y.M.);

**Keywords:** anti-inflammation, sweet potato (*Ipomoea batatas* (L.), Nrf2, RAW264.7

## Abstract

Sweet potato is a crop that is widely consumed all over the world and is thought to contribute to health maintenance due to its abundant nutrients and phytochemicals. Previous studies on the functionality of sweet potatoes have focused on varieties that have colored pulp, such as purple and orange, which contain high levels of specific phytochemicals. Therefore, in the present study, we evaluated the anti-inflammatory effects of light-yellow-fleshed sweet potatoes, which have received little attention. After freeze-drying sweet potatoes harvested in 2020, extracts were prepared from the leaves, stems, roots, and tubers in 100% ethanol. Mouse macrophage-like cell line RAW264.7 cells were cultured with 10 µg/mL of the extracts and induced lipopolysaccharide (LPS)-stimulated inflammation. Of the extracts, the tuber extracts showed the highest suppression of LPS-induced interleukin-6 (IL-6) gene expression and production in RAW264.7, which was attributed to the activation of the nuclear factor erythroid 2-related factor 2 (Nrf2) oxidative stress response pathway. In addition, preparative high-performance liquid chromatography (HPLC) experiments suggested that hydrophobic components specific to the tuber were the main body of activity. In previous studies, it has been shown that the tubers and leaves of sweet potatoes with colored pulp exhibit anti-inflammatory effects due to their rich phytochemicals, and our results show that the tubers with light-yellow pulp also exhibit the effects. Furthermore, we were able to show a part of the mechanism, which may contribute to the fundamental understanding of the treatment and prevention of inflammation by food-derived components.

## 1. Introduction

Inflammation is a defense response in the body, and macrophages play a role in this response by phagocytosing targets and secreting molecules such as nitric oxide, cytokines, and chemokines, thereby activating the immune response. Although inflammation is, in essence, a response to protect the organism, various studies in recent years have shown that chronic inflammation causes various problems in the tissues of the body and is closely related to the onset and worsening of age-related diseases, such as diabetes and cancer [1,2,3]. Although the most effective treatment for such chronic inflammation is probably the suppression of inflammation with drugs, from the perspective of social problems, such as increased medical costs, we should also pay attention to improvements in symptoms due to food-derived ingredients. It is believed that food-derived ingredients with anti-inflammatory effects can prevent or treat diseases, and there have been many reports on their effects, including one that examined the relationship between dietary flavonoid intake and the risk of developing chronic diseases [4,5]. In that report, it was mentioned that quercetin and other flavonoids may reduce the risk of heart disease and type II diabetes mellitus.

The anti-inflammatory effects of food-derived ingredients are often related to their antioxidant properties [5,6]. When inflammatory signals are activated in macrophages, reactive oxygen species (ROS) are generated, which are involved in signal transduction as secondary messengers, and play important roles in metabolism and proliferation; however, they can also cause oxidative stress and damage DNA and cells [7]. Some food-derived components are reported to have antioxidant effects by scavenging ROS and activating oxidative stress response pathways [8,9,10].

NF-E2-related factor 2 (Nrf2) is a key transcription factor that plays a central role in the oxidative stress response pathway. Its function is inhibited under normal conditions, as it binds to Kelch-like ECH-associated protein 1 (Keap1). It is released and translocated into the nucleus upon activation, and it recognizes antioxidant response element (ARE) sequences that activate the transcription of antioxidant factors such as Heme Oxygenase 1 (HO-1) and NAD(P)H quinone oxidoreductase 1 (NQO-1) [11]. In addition to suppressing inflammatory responses by reducing ROS through HO-1 and other functions, it has also been reported to inhibit NF-κB, which transduces inflammatory signals [12,13,14].

Sweet potato is a perennial herbaceous plant found in the *Ipomoea* genus of the Hirudoaceae family; it is drought tolerant and produces high yields, even on barren land. Globally, it is mostly produced in China and African regions, but its tuberous roots are eaten in many parts of the world and are expected to have a positive effect on health due to their high dietary fiber, vitamin, and mineral content [15]. There is diversity in pulp color among cultivars, which are mainly light yellow, orange, and purple. The colored cultivars are rich in phytochemicals, with the orange ones containing more carotenoids and the purple ones containing more anthocyanin [16,17]. These colored sweet potatoes have various functional properties, and there have been many reports dedicated to their relationship with the immune system, which has attracted much attention in recent years [18,19,20]. Sugata et al. reported that anthocyanin in purple sweet potatoes reduced the production of lipopolysaccharide (LPS)-induced inflammatory cytokines (tumor necrosis factor α(TNFα), interleukin-6 (IL-6), and nitric oxide (NO)) in RAW264.7 cells and inhibited the growth of several cancer cell lines. Similarly, Bae et al. reported that β-carotene in orange sweet potatoes also inhibited LPS-induced production of IL-6, NO, and prostaglandin E2 (PGE2) in RAW 264.7. However, these colored sweet potatoes are still not commonly eaten, at least in Japan, and reports on the functional properties of light-yellow-fleshed cultivars, which are mainly consumed, are still poor. In order to benefit from functional food components, it is important that they be found in commonly consumed foods. Therefore, the present study focused on the light-yellow cultivar Beniharuka, whose tubers are widely consumed in Japan, and verified whether it also has the anti-inflammatory effects that have been confirmed with colored sweet potatoes. In the results, we found that extracts from tuberous roots of light-yellow pulp sweet potato suppressed the expression of pro-inflammatory genes via the Nrf2 oxidative stress response pathway.

## 2. Materials and Methods

### 2.1. Preparation of Sweet Potato Samples and Extracts

The sweet potatoes used in this study were sampled from Ibaraki, Japan, in July 2020. Samples were collected from 10 plants, washed immediately in water, and the leaves, stems, roots, and tuberous roots were separated and freeze-dried. The samples were then pulverized and extracted using 100% ethanol (Kanto Chemical, Tokyo, Japan); the solvent was evaporated in an evaporator and suspended in dimethyl sulfoxide (DMSO; Fujifilm Wako Pure Chemicals, Osaka, Japan) to create a 10 mg/mL extract.

### 2.2. Reagents and Antibodies

LPS (*Escherichia*. *coli* O127) was purchased from Fujifilm Wako Pure Chemicals (Osaka, Japan). Anti-Lamin B1 (ab16048), anti-Nrf2 (ab2352), and anti-HO-1 (ab68477) were from Abcam (Cambridge, UK), and anti-NF-κB (#8242), anti-β-Actin (#4967) and anti-rabbit IgG-HRP (#7074) were purchased from Cell Signaling Technology (Danvers, MA). Nrf2 inhibitor ML385 was purchased from Selleck (Houston, TX, USA).

### 2.3. Cell Culture and Stimulation Conditions

RAW264.7 murine macrophage cells were obtained from KAC (Kyoto, Japan). Cells were cultured in Dulbecco’s Modified Eagle Medium (DMEM; Sigma-Aldrich, St. Louis, MO, USA) plus 10% deactivated fetal bovine serum (FBS; Biowest, Nuaillé, France) and 1% penicillin–streptomycin (Gibco, Grand Island, NY, USA) at 37 °C in 5% CO_2_. Extracts were added to the medium at a final concentration of 10 µg/mL (1000×) and incubated for 1 h, after which LPS (final concentration 50 ng/mL) was added. Unless otherwise mentioned, these conditions were standardized for all cell cultures and stimulation.

### 2.4. Cell Viability Assay

RAW264.7 cells were cultured in 96-well plates (AGC Technoglass, Shizuoka, Japan) at a density of 1.0 × 10^4^ cells/well for 24 h and each extract was added at final concentrations of 1, 10, and 100 µg/mL. After 1 h of incubation, LPS stimulation was performed, and the cells were incubated for 24 h. Then, the WST-1 reagent (Cayman Chemical, Ann Arbor, MI, USA) was added, and the cells were incubated for another 2 h. Finally, the absorbance of the culture supernatant at 450 nm was measured by a microplate reader (model 680, Bio-rad, Hercules, CA, USA) to determine the cell survival rate.

### 2.5. Enzyme-Linked Immunosorben Assay (ELISA)

RAW264.7 cells were cultured in 96-well plates at a density of 1.0 × 10^4^ cells/well. After 24 h of incubation, each extract and LPS were added. After incubation for another 24 h, the supernatant was collected and used for the assay. An ELISA MAX™ Deluxe Set Mouse IL-6 (BioLegend, San Diego, CA, USA) was used for the experiments, following the described protocol.

### 2.6. Intracellular ROS Observation

RAW264.7 cells were cultured in 96-well plates at a density of 1.0 × 10^4^ cells/well for 24 h, and then each extract and LPS were added. After incubation for another 20 h, intracellular ROS was stained using an ROS Assay Kit-Highly Sensitive DCFH-DA (Dojindo Laboratories, Kumamoto, Japan) and observed using a fluorescence microscope (BZ-X810, Keyence, Osaka, Japan).

### 2.7. RT-qPCR

RAW264.7 cells were cultured in 96-well plates at a density of 1.0 × 10^4^ cells/well, and each extract and LPS were added after 24 h of culture. After another 24 h of culture, cells were collected, and cDNA was prepared using the SuperPrep^®^ Cell Lysis & RT Kit for qPCR (TOYOBO, Osaka, Japan). RT-qPCR was performed using THUNDERBIRD^®^ Next SYBR^®^ qPCR Mix (TOYOBO) and QuantStudio 5 real-time PCR system (Thermo Fisher Scientific, Waltham, MA, USA), and the relative expression levels between samples were evaluated using the ΔΔCt method. Primer information is shown in Table 1.

### 2.8. Western Blotting

RAW264.7 cells were cultured in 60 mm tissue culture dishes (AGC Technoglass, Shizuoka, Japan) at a density of 2.0 × 10^5^ cells/dish, and extracts and LPS were added after 24 h. After LPS stimulation, the cells were sampled at predetermined time points. 

For whole-cell sampling, cells were lysed in 250 μL lysis buffer (50 mM Tris-HCl (pH 7.5), 150 mM NaCl, 0.5% sodium dodecyl sulfate (SDS), protease inhibitor cocktail (cOmplete™ Mini, Roche, Basel, Switzerland), and phosphatase inhibitor cocktail (PhosSTOP™, Roche, Basel, Switzerland), and the suspension was incubated on ice for at least 1 h. The supernatant obtained from centrifugation (20,000× *g*, 15 min, 4 °C) was mixed with 4× Laemmli’s sample buffer (Bio-rad, Hercules, CA, USA) to make samples.

For separation of the nuclear and cytoplasmic fractions, cells were lysed in 200 µL cytoplasmic lysis buffer (10 mM HEPES (pH 7.9), 10 mM KCl, 1.5 mM MgCl_2_, 1 mM dithiothreitol (DTT), 0.5% NP-40, protease inhibitors, and phosphatase inhibitors). The suspension was incubated on ice for at least 15 min, and the supernatant was centrifuged (800× *g*, 10 min, 4 °C) to obtain the cytoplasmic fraction. The precipitated pellet was dissolved in 100 µL nucleolytic buffer (20 mM HEPES (pH 7.9), 400 mM NaCl, 1.5 mM MgCl_2_, 0.2 mM EDTA, 1 mM DTT, 10% Glycerol, protease inhibitors, phosphatase inhibitors). The supernatant was then sonicated on ice and centrifuged (14,000× *g*, 30 min, 4 °C) to obtain the nuclear fraction. Each was mixed with 4× Laemmli’s sample buffer to form the separated samples.

SDS-PAGE was performed on the prepared samples, which were transferred to Immobilon-P PVDF membranes (Merck, Darmstadt, Germany). The target proteins on the PVDF membrane were then labeled with antibodies, detected with SuperSignal™ West Femto Maximum Sensitivity Substrate (Thermo Fisher Scientific), and analyzed using a Lumino Image Analyzer (ImageQuant LAS 800, Cytiva, Marlborough, MA, USA). The intensity of each band was analyzed using ImageJ software (Ver. 1.53).

### 2.9. High-Performance Liquid Chromatography (HPLC)

Analysis of the properties of the components contained in the extract was performed with an Agilent 1260 Infinity (Agilent Technologies, Santa Clara, CA, USA) ODS column (Cadenza CD-C18 column, 250 × 4.6 mm, particle size 3 µm, Imtakt Corporation, Kyoto, Japan) at a constant temperature of 40 °C. In the mobile phase, a 5-fold-diluted extract in ethanol of 30 µL was applied to the column, and elution was performed at a flow rate of 1 mL/min with a mixture of solvents A (0.4% formic acid (Fujifilm Wako Pure Chemicals, Osaka, Japan)) and B (100% acetonitrile (Kanto Chemical, Tokyo, Japan)). Elution was performed under the following conditions: gradient from initial conditions to 40 min (0 min (A:B = 93:7), 33 min (A:B = 60:40), 40 min (A:B = 0:100)), from 40 to 70 min (A:B = 0:100). Spectra were detected with a diode array detector (DAD) at 280 and 326 nm. In parallel with the analysis, fraction collection was also performed. The preparative schedule was as follows: Fr.1: 8–15 min, Fr.2: 19–25 min, Fr.3: 42–51 min, Fr.4: 51–60 min, Fr.5: 60–69 min.

### 2.10. DPPH Radical Scavenging Activity Assay

To a 96-well plate, 50 µL of Trolox standard solution, a sweet potato extract sample (5 mg/mL in 50% ethanol) and a blank (50% ethanol) were added. Then, 150 µL of 200 µM 1,1-diphenyl-2-picrylhydrazyl (DPPH) solution was added to each well and agitated for 15 min. The absorbance at 540 nm was then measured using a microplate reader, and the DPPH radical scavenging activity per 100 g of sample was calculated as µmol Trolox equivalent.

### 2.11. Total Polyphenol Content Assay

As standard solutions, gallic acid was adjusted to 500, 250, 100, 50, and 25 µg/mL. To a test tube, 100 µL of each concentration of gallic acid standard or each sweet potato extract (10 mg/mL in 100% ethanol) was added, followed by 2 mL of distilled water. After adding 500 µL of Folin and Ciocalteu’s phenol reagent (MP Biomedicals, Santa Ana, CA, USA), 500 µL of a 10% sodium carbonate solution was quickly added, mixed, and placed in the dark for 1 h at room temperature. The absorbance at 760 nm was measured, and the total polyphenol content per 100 g of sample was calculated.

### 2.12. Total Flavonoid Content Assay

As standard solutions, catechin hydrate was prepared at concentrations of 500, 250, 100, 50, and 25 µg/mL. To the test tubes, 250 µL of each concentration of catechin standard solution or sweet potato extract (10 mg/mL in 100% ethanol) was added. They were mixed with 1250 µL of distilled water and 75 µL of 5% sodium nitrite solution. After 5 min of incubation, 150 µL of 10% aluminum chloride solution was added and mixed. After another 6 min of incubation, 500 µL of 1 M sodium hydroxide solution and 275 µL of distilled water were added and mixed. The absorbance at 510 nm was then measured, and the total flavonoid content per 100 g of sample was calculated.

### 2.13. Statistical Analysis

Statistical analysis was performed with GraphPad Prism 9 (MDF, Tokyo, Japan). A Student’s *t*-test was performed to compare the two groups, and one-way analysis of variance (ANOVA) was performed to test three or more groups, followed by Dunnett’s or Tukey’s post-hoc test. The significance level was set at 5%.

## 3. Results

### 3.1. Sweet Potato Tuber Extracts Suppressed the Production of Inflammatory Cytokines and Gene Expression

**Figure 1:**
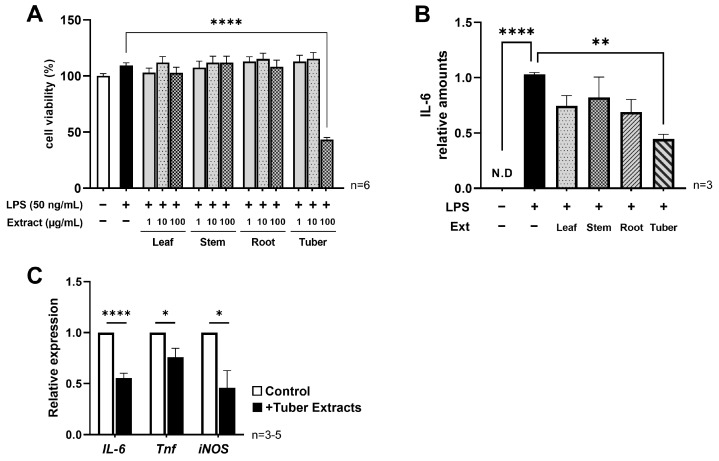
Cell viability and expression of LPS-induced pro-inflammatory cytokines under conditions with sweet potato extracts: (**A**) Cell viability at 24 h of incubation after the addition of the sweet potato extracts at concentrations of 1 µg/mL, 10 µg/mL, and 100 µg/mL; (**B**) Relative production level of IL-6 at 24 h after LPS stimulation with/without each sweet potato extracts; (**C**) Relative expression of pro-inflammatory genes (*IL-6*, *Tnf*, *iNOS*(*Nos2*)) at 24 h after LPS stimulation with/without sweet potato tuber extracts: (**A**,**B**) mean ± SEM, one-way ANOVA with Dunnett’s post-hoc test (vs +LPS), **: *p* < 0.01, ****: *p* < 0.0001; (**C**) mean ± SEM, Student’s *t*-test, *: *p* < 0.05, ****: *p* < 0.0001: LPS, lipopolysaccharide; Ext, extract.

The effects of extracts of sweet potato leaf, stem, root, and tuber on the viability of RAW264.7 cells under LPS-stimulated conditions were determined. Up to a concentration of 10 µg/mL, no extract showed any change in viability, but at 100 µg/mL, the tuber extract showed a significant decrease in viability (Figure 1A). Therefore, we set 10 µg/mL as the prescribed concentration of the extracts in subsequent experiments. IL-6 is one of the main inflammatory markers whose expression is increased by LPS stimulation in macrophages, and we selected it as an indicator of inflammatory intensity in this study. Cells were stimulated in the presence of sweet potato extract, and the IL-6 concentration in the medium at 24 h after stimulation was significantly reduced by about 50% in the presence of the tuber extract (Figure 1B). The expression of the *IL-6* gene was also suppressed, as well as that of other proinflammatory genes such as *Tnf* and *iNOS* (Figure 1C). These results indicate that only the tuber extract suppressed LPS-induced inflammatory responses.

### 3.2. Tuber Extracts Induce Activation of the Nrf2 Oxidative Stress Response Pathway and Inhibit the Function of NF-κB

**Figure 2:**
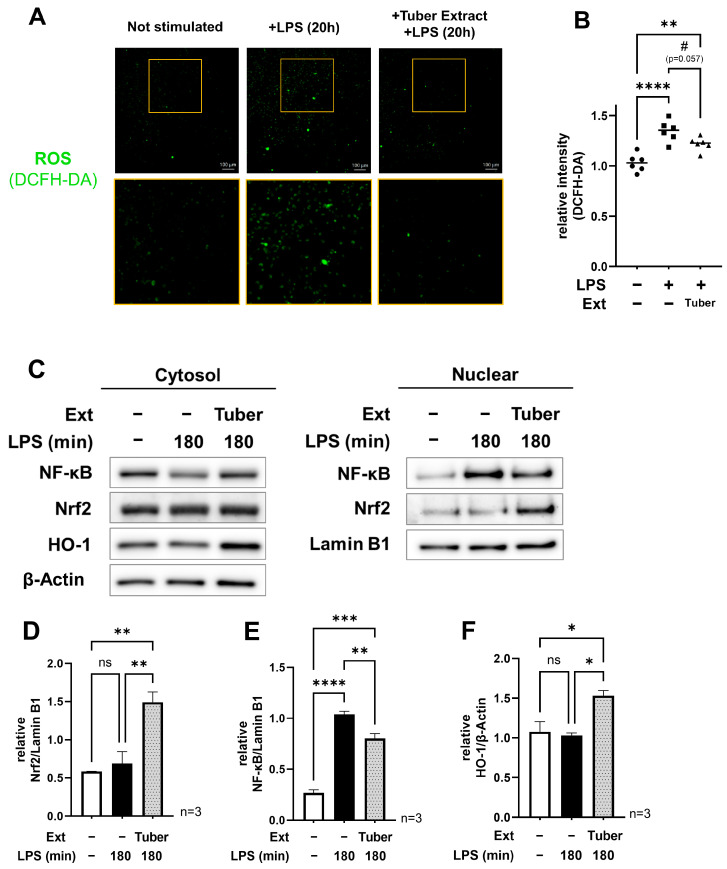
Analysis of intracellular ROS, NF-κB, and Nrf2 oxidative stress response pathway under tuber extract conditions: (**A**,**B**) Fluorescence microscopy images of intracellular ROS at 20 h after LPS stimulation with/without tuber extracts and comparison of its fluorescence intensity per cell; (**C**) Western blot of nuclear and cytoplasmic fractions at 180 min after LPS stimulation in the presence of tuber extracts; (**D**–**F**) Relative quantification of nuclear NF-κB, Nrf2, and cytoplasmic HO-1: mean ± SEM, one-way ANOVA with Tukey’s post-hoc test, #: *p* < 0.1, *: *p* < 0.05, **: *p* < 0.01, ***: *p* < 0.001, ****: *p* < 0.0001, ns: not significant : LPS, lipopolysaccharide; Ext, extract; ROS, reactive oxygen species.

We observed intracellular ROS using fluorescence microscopy and quantified the fluorescence intensity of each cell after 20 h of LPS stimulation in the presence of extracts of sweet potato tubers and found that the addition of the tuber extracts tended to decrease ROS (Figure 2A,B). Among the four extracts used in this study, the highest values for total polyphenols, total flavonoids, and DPPH radical scavenging activity were found in the leaf extracts, and the other three extracts had significantly lower values (Figure S1). Thus, in the present study, the high antioxidant capacity of the extracts did not simply act to suppress inflammation. These results suggest that the tuber extract induced the activation of an intracellular antioxidant function. Therefore, we focused on Nrf2, which is known to be an oxidative stress response factor. We used Western blotting to examine nuclear Nrf2 180 min after LPS stimulation and found that nuclear Nrf2 increased with tuber extract addition (Figure 2C,D). At the same time, nuclear NF-κB was reduced (Figure 2E). We also found an increase in downstream-regulated HO-1 in the cytoplasmic fraction (Figure 2F), suggesting that the tuber extract somehow activates the Nrf2 pathway and suppresses the expression of pro-inflammatory genes through NF-κB.

### 3.3. Nrf2 Inhibition Cancels the Anti-Inflammatory Effect of the Tuber Extracts

**Figure 3:**
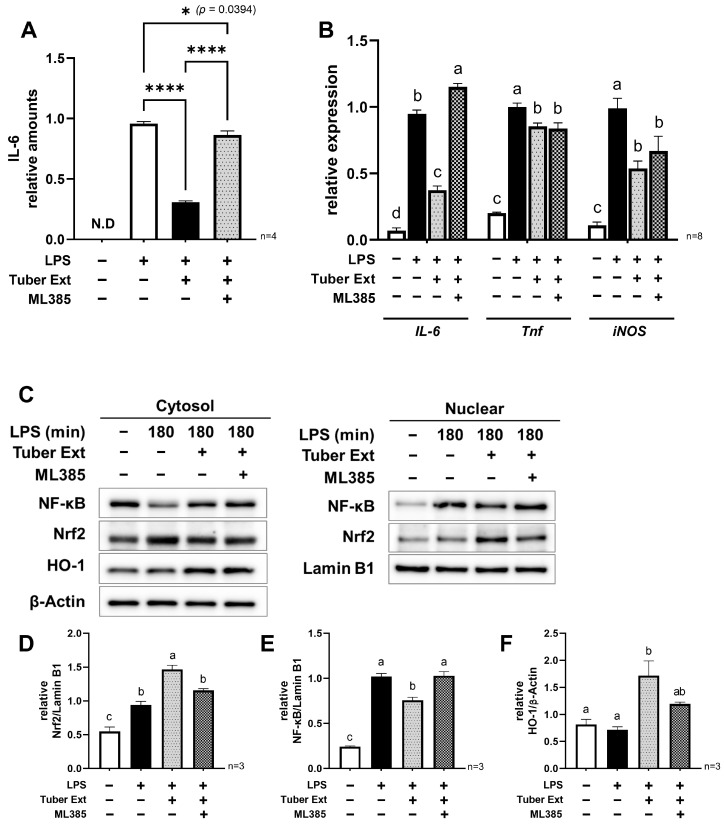
Analysis of the effects of Nrf2 inhibition on the suppression of LPS-induced inflammation by tuber extracts: (**A**) Relative production level of IL-6 at 24 h after LPS stimulation with/without tuber extracts and ML385; (**B**) Relative expression of pro-inflammatory genes (*IL-6*, *Tnf*, *iNOS*(*Nos2*)) at 24 h after LPS stimulation with/without tuber extracts and ML385; (**C**) Western blot of nuclear and cytoplasmic fractions at 180 min after LPS stimulation in the presence of tuber extracts and ML385; (**D**–**F**) Relative quantification of nuclear NF-κB, Nrf2 and cytoplasmic HO-1 under each condition: (**A**,**B**,**D**–**F**) mean ± SEM, one-way ANOVA with Tukey’s post-hoc test, *: *p* < 0.05, ****: *p* < 0.0001, Distinct symbols (a, b, c, d) are used to denote significant differences between groups: LPS, lipopolysaccharide; Ext, extract.

To verify the involvement of Nrf2 in the suppression of inflammation-related gene expression by tuber extracts, we conducted experiments using ML385, a small molecule compound known as an Nrf2 inhibitor. ML385 binds to the CNC-bZIP domain of Nrf2, Neh1, thereby blocking its ability to bind to the small molecule protein Maf and inhibiting the activity of Nrf2 as a transcription factor [21]. We conducted an experiment in which the inhibitor ML385 was added to the medium 1 h before the addition of tuber extract and evaluated LPS-induced IL-6 production, the expression of inflammation-related genes, and the nuclear translocation of Nrf2 and NF-κB. As a result, the concentration of IL-6 in the medium after 24 h of LPS stimulation with ML385 was almost the same as that without the extract (Figure 3A). Furthermore, we confirmed that ML385 canceled out the extract-induced decrease in *IL-6* gene expression (Figure 3B). However, no significant changes were observed in the expression of other inflammation-related genes (*Tnf* and *iNOS*), suggesting that the decreased expression of these genes was regulated by a different pathway. Under the conditions with added ML385, nuclear Nrf2, NF-κB, and cytoplasmic HO-1 levels were similar to those without the extract, suggesting that NF-κB’s function was inhibited by the activation of the Nrf2 pathway (Figure 3C–F). These results for the addition of ML385 suggest that the components in the sweet potato tuber inhibited the function of NF-κB via the activation of the Nrf2 pathway, leading to the suppression of LPS-induced IL-6 expression.

### 3.4. Hydrophobic Components in the Tuber Contribute to Its Anti-Inflammatory Properties

**Figure 4:**
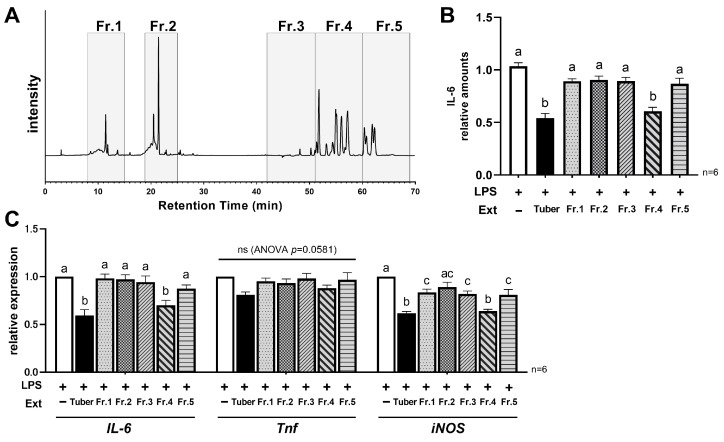
Characterization of the components responsible for anti-inflammatory activity in tuber extracts: (**A**) Peaks observed in HPLC analysis of tuber extracts and fraction settings; (**B**) Relative production level of IL-6 at 24 h after LPS stimulation with/without tuber extracts or each fraction; (**C**) Relative expression of pro-inflammatory genes (*IL-6*, *Tnf*, *iNOS*(*Nos2*)) at 24 h after LPS stimulation with/without tuber extracts or each fraction: (**B**,**C**) mean ± SEM, one-way ANOVA with Tukey’s post-hoc test, Distinct symbols (a, b, c) are used to denote significant differences between groups: LPS, lipopolysaccharide; Ext, extract; Fr., fraction.

To characterize the active components in the extract, we fractionated the extract based on high-performance liquid chromatography (HPLC) analysis. HPLC analysis revealed multiple peaks, both under elution with 0.4% formic acid and with acetonitrile alone (Figure 4A). Based on the data we obtained, the extracts were collected in five fractions (Fr.1–Fr.5), as shown in Figure 4A. After drying and solidifying, each fraction was resuspended to a concentration equivalent to that of the original extract. In the IL-6 ELISA assay, a similar level of reduction was observed under conditions with added Fr.4 as in the original tuber extract (Figure 4B). In addition, among the five fractions, the addition of Fr.4 resulted in the greatest reduction in *IL-6* and *iNOS* expression (Figure 4C). The HPLC conditions for eluting Fr.4 were “0.4% formic acid:acetonitrile = 0:100”, suggesting that the main component in Fraction 4 is a hydrophobic component. Moreover, when extracts of leaves, stems, and roots were analyzed by HPLC under the same conditions, most of the peaks in these fractions were undetectable (Figure S2). Therefore, the hydrophobic component that is only contained within the tuber may contribute to the anti-inflammatory effect confirmed in this study.

## 4. Discussion

In this study, we evaluated the anti-inflammatory activity of different parts of light-yellow pulp sweet potato, which have received little attention thus far. Among the leaves, stems, roots, and tubers, only extracts from tubers, the edible part of the plant, showed significant anti-inflammatory activity. Furthermore, the suppression of IL-6 production and expression was shown to be mediated by the activation of the Nrf2 pathway. It was also suggested that the hydrophobic component of the tuber extract contributed to its anti-inflammatory effects.

Previous studies have shown that many of the anti-inflammatory effects of foods or plants are associated with components with high antioxidant capacity [5,6]. It has been reported that anthocyanins in purple sweet potatoes and β-carotene in orange sweet potatoes inhibit LPS-induced inflammatory cytokine production in RAW264.7 cells [18,19]. Thus, phytochemicals with high antioxidant capacities are known to be involved in anti-inflammation in vitro. It should be noted, however, that high antioxidant capacity does not mean high anti-inflammatory activity. In the present study, the leaf extract had the highest values of total polyphenols, total flavonoids, and DPPH radical scavenging activity among the four extracts (Figure S1). Makori et al. also reported that among the parts of several cultivars of sweet potatoes, the leaves contain the highest amount of polyphenols and flavonoids, and exhibit high antioxidant activity [22]. In their report, they also showed that the same tendency was observed in purple and orange sweet potatoes. Nevertheless, in the present study, we found that the expression of inflammatory cytokines is more suppressed with tuber extracts. In addition, as shown in Figure 4, most of the peaks in the hydrophobic fraction, which is thought to play a central role in the anti-inflammatory activity, were detected in the tuber but not in the other three extracts (Figure S2). Therefore, these suggest that the key to anti-inflammation is not the antioxidant capacity of the extract, but the components it contains and that the component contributing to the anti-inflammatory activity is likely to be present only in the tubers in sweet potatoes. Although we were unable to identify the components responsible for this activity in this study, previous studies have reported that some carotenoids, flavonoids, and lipid-soluble polyphenols are contained in sweet potato tuber [23,24,25], which may be related to our findings. For instance, caffeic acid ethyl ester, one of the lipid-soluble polyphenols, has been reported to exhibit anticancer effects [25], and a number of other components are expected to have such activity via biochemical mechanisms. Identifying the components is an important issue to be addressed in future research, as it is a key factor in applying our findings to the prevention and improvement of chronic inflammation.

The present study also indicated that the activation of the Nrf2 pathway is, at least, involved in the suppression of *IL-6* gene expression. We hypothesize that the increased nuclear translocation of Nrf2 and the function of downstream factors contribute to this. First, the promoter region upstream of the *IL-6* gene contains a common binding site for Nrf2 and NF-κB, and Nrf2 binding in this site inhibits the function of Pol II [14], suggesting that the increased nuclear translocation of activated Nrf2 simply reduced IL-6 expression. We also observed increased expression of the downstream factor (Figure 2C,F). As HO-1 and NQO-1, which are downstream factors, have been reported to inhibit the NF-κB signaling pathway [12,26], we believe that this indirectly led to the suppression of inflammation-related gene expression. There are many reports of anti-inflammatory effects mediated by Nrf2 activation. Some of them have been linked to medicinal plants or herbs, such as Chinese sweet plum (*Sageretia thea* (Osbek.)), Ginseng (*Penax ginseng* (C. A. Mey.)), Elecampane (*Inula helenium* (L.)) [27,28,29], and other components in commonly consumed foods, such as pyrocatechol in roasted coffee [10], indicating that effects on daily food intake may also be expected. 

In this study, we showed that components in light-yellow pulp sweet potato tuber extract suppressed LPS-induced inflammation in RAW264.7 cells via the Nrf2 pathway, but the specific components responsible for the activity, upstream of Nrf2 activation, and in vivo effects remain unclear. However, sweet potato is a daily consumed crop in many regions, and these results may help to encourage consumption as a dietary approach against inflammation. In the future, we plan to examine the effects of heat processing on the activity.

## 5. Conclusions

While it has been known that sweet potatoes with purple or orange pulp have anti-inflammatory effects due to the plant metabolites contained abundantly in them, we described the anti-inflammatory effects of light-yellow pulp sweet potatoes in this study. We found that only extracts from the tuber, among the four parts of the plant (leaf, stem, root, and tuber), suppressed LPS-induced inflammation in RAW264.7 cells, and this effect may be attributed to the inhibition of NF-κB associated with the activation of the Nrf2 pathway. It was also shown that the hydrophobic components of the tuber are responsible for the activity, but the specific components remained unknown within this study and remain an issue to be addressed in the future. However, we believe that these results expand the potential of sweet potato as a food-derived approach to inflammation.

## Figures and Tables

**Table 1:** Primer information.

Gene	Primer Sequence	
*IL-6*	Forward:	AGAGGATACCACTCCCAACAGA
	Reverse:	CTGCAAGTGCATCATCGTTGTTC
*Tnf*	Forward:	ATTCGAGTGACAAGCCTGTAG
	Reverse:	TGAAGAGAACCTGGGAGTAGAC
*iNOS (Nos2)*	Forward:	AGGCTGGAAGCTGTAACAAAGG
	Reverse:	GCTGAAACATTTCCTGTGCTGTG
*Actb*	Forward:	ACTATTGGCAACGAGCGGTTC
	Reverse:	TCAGCAATGCCTGGGTACATG

## Data Availability

The raw data supporting the conclusions of this article will be made available by the authors on request.

## Supplementary Materials

The following supporting information can be downloaded at: https://www.mdpi.com/article/10.3390/nu16040563/s1, Figure S1: Total polyphenols, total flavonoids, and DPPH radical scavenging activity of sweet potato extracts; Figure S2: Comparison of peaks observed in HPLC analysis of sweet potato extracts.

## References

[1] Franceschi C., Campisi J. (2014). Chronic inflammation (inflammaging) and its potential contribution to age-associated diseases. J. Gerontol. A Biol. Sci. Med. Sci..

[2] Boutens L., Hooiveld G.J., Dhingra S., Cramer R.A., Netea M.G., Stienstra R. (2018). Unique metabolic activation of adipose tissue macrophages in obesity promotes inflammatory responses. Diabetologia.

[3] Giallongo C., Tibullo D., Camiolo G., Parrinello N.L., Romano A., Puglisi F., Barbato A., Conticello C., Lupo G., Anfuso C.D. (2019). TLR4 signaling drives mesenchymal stromal cells commitment to promote tumor microenvironment transformation in multiple myeloma. Cell Death. Dis..

[4] Knekt P., Kumpulainen J., Jarvinen R., Rissanen H., Heliovaara M., Reunanen A., Hakulinen T., Aromaa A. (2002). Flavonoid intake and risk of chronic diseases. Am. J. Clin. Nutr..

[5] Rudrapal M., Khairnar S.J., Khan J., Dukhyil A.B., Ansari M.A., Alomary M.N., Alshabrmi F.M., Palai S., Deb P.K., Devi R. (2022). Dietary Polyphenols and Their Role in Oxidative Stress-Induced Human Diseases: Insights Into Protective Effects, Antioxidant Potentials and Mechanism(s) of Action. Front. Pharmacol..

[6] Maleki S.J., Crespo J.F., Cabanillas B. (2019). Anti-inflammatory effects of flavonoids. Food. Chem..

[7] Schieber M., Chandel N.S. (2014). ROS function in redox signaling and oxidative stress. Curr. Biol..

[8] Na H.K., Surh Y.J. (2008). Modulation of Nrf2-mediated antioxidant and detoxifying enzyme induction by the green tea polyphenol EGCG. Food Chem. Toxicol..

[9] Cardozo L.F., Pedruzzi L.M., Stenvinkel P., Stockler-Pinto M.B., Daleprane J.B., Leite M., Mafra D. (2013). Nutritional strategies to modulate inflammation and oxidative stress pathways via activation of the master antioxidant switch Nrf2. Biochimie.

[10] Funakoshi-Tago M., Nonaka Y., Tago K., Takeda M., Ishihara Y., Sakai A., Matsutaka M., Kobata K., Tamura H. (2020). Pyrocatechol, a component of coffee, suppresses LPS-induced inflammatory responses by inhibiting NF-kappaB and activating Nrf2. Sci. Rep..

[11] Bellezza I., Giambanco I., Minelli A., Donato R. (2018). Nrf2-Keap1 signaling in oxidative and reductive stress. Biochim. Biophys. Acta Mol. Cell. Res..

[12] Willis D., Moore A.R., Frederick R., Willoughby D.A. (1996). Heme oxygenase: A novel target for the modulation of the inflammatory response. Nat. Med..

[13] Liu G.H., Qu J., Shen X. (2008). NF-kappaB/p65 antagonizes Nrf2-ARE pathway by depriving CBP from Nrf2 and facilitating recruitment of HDAC3 to MafK. Biochim. Biophys. Acta.

[14] Kobayashi E.H., Suzuki T., Funayama R., Nagashima T., Hayashi M., Sekine H., Tanaka N., Moriguchi T., Motohashi H., Nakayama K. (2016). Nrf2 suppresses macrophage inflammatory response by blocking proinflammatory cytokine transcription. Nat. Commun..

[15] Oin Y., Naumovski N., Ranasheera C.S., D’Cunha N.M. (2022). Nutrition-related health outcomes of sweet potato (*Ipomoea batatas*) consumption: A systematic review. Food Biosci..

[16] Islam S.N., Nusrat T., Begum P., Ahsan M. (2016). Carotenoids and beta-carotene in orange fleshed sweet potato: A possible solution to vitamin A deficiency. Food Chem..

[17] Chen C.C., Lin C., Chen M.H., Chiang P.Y. (2019). Stability and Quality of Anthocyanin in Purple Sweet Potato Extracts. Foods.

[18] Sugata M., Lin C.Y., Shih Y.C. (2015). Anti-Inflammatory and Anticancer Activities of Taiwanese Purple-Fleshed Sweet Potatoes (*Ipomoea batatas* L. Lam) Extracts. Biomed Res. Int..

[19] Bae J.Y., Park W.S., Kim H.J., Kim H.S., Kang K.K., Kwak S.S., Ahn M.J. (2021). Protective Effect of Carotenoid Extract from Orange-Fleshed Sweet Potato on Gastric Ulcer in Mice by Inhibition of NO, IL-6 and PGE(2) Production. Pharmaceuticals.

[20] Jiang T., Zhou J., Liu W., Tao W., He J., Jin W., Guo H., Yang N., Li Y. (2020). The anti-inflammatory potential of protein-bound anthocyanin compounds from purple sweet potato in LPS-induced RAW264.7 macrophages. Food Res. Int..

[21] Singh A., Venkannagari S., Oh K.H., Zhang Y.Q., Rohde J.M., Liu L., Nimmagadda S., Sudini K., Brimacombe K.R., Gajghate S. (2016). Small Molecule Inhibitor of NRF2 Selectively Intervenes Therapeutic Resistance in KEAP1-Deficient NSCLC Tumors. ACS Chem. Biol..

[22] Makori S., Mu T.-H., Sun H.-N. (2020). Total Polyphenol Content, Antioxidant Activity, and Individual Phenolic Composition of Different Edible Parts of 4 Sweet Potato Cultivars. Nat. Prod. Commun..

[23] Ishiguro K., Yoshinaga M., Kai Y., Maoka T., Yoshimoto M. (2010). Composition, content and antioxidative activity of the carotenoids in yellow-fleshed sweetpotato (*Ipomoea batatas* L.). Breed. Sci..

[24] Wang A., Li R., Ren L., Gao X., Zhang Y., Ma Z., Ma D., Luo Y. (2018). A comparative metabolomics study of flavonoids in sweet potato with different flesh colors (*Ipomoea batatas* (L.) Lam). Food Chem..

[25] Kato K., Nagane M., Aihara N., Kamiie J., Miyanabe M., Hiraki S., Luo X., Nakanishi I., Shoji Y., Matsumoto K.I. (2021). Lipid-soluble polyphenols from sweet potato exert antitumor activity and enhance chemosensitivity in breast cancer. J. Clin. Biochem. Nutr..

[26] Kimura A., Kitajima M., Nishida K., Serada S., Fujimoto M., Naka T., Fujii-Kuriyama Y., Sakamato S., Ito T., Handa H. (2018). NQO1 inhibits the TLR-dependent production of selective cytokines by promoting IkappaB-zeta degradation. J. Exp. Med..

[27] Yang S., Li F., Lu S., Ren L., Bian S., Liu M., Zhao D., Wang S., Wang J. (2022). Ginseng root extract attenuates inflammation by inhibiting the MAPK/NF-kappaB signaling pathway and activating autophagy and p62-Nrf2-Keap1 signaling in vitro and in vivo. J. Ethnopharmacol..

[28] Kim H.N., Park G.H., Park S.B., Kim J.D., Eo H.J., Son H.J., Song J.H., Jeong J.B. (2019). Sageretia thea Inhibits Inflammation through Suppression of NF- kappa B and MAPK and Activation of Nrf2/HO-1 Signaling Pathways in RAW264.7 Cells. Am. J. Chin. Med..

[29] Park E.J., Kim Y.M., Park S.W., Kim H.J., Lee J.H., Lee D.U., Chang K.C. (2013). Induction of HO-1 through p38 MAPK/Nrf2 signaling pathway by ethanol extract of *Inula helenium* L. reduces inflammation in LPS-activated RAW 264.7 cells and CLP-induced septic mice. Food Chem. Toxicol..

