# Supplementary material for: Hydrophobic Components in Light-Yellow Pulp Sweet Potato (Ipomoea batatas (L.) Lam.) Tubers Suppress LPS-Induced Inflammatory Responses in RAW264.7 Cells via Activation of the Nrf2 Pathway
Source: Nutrients. 2024 Feb 18;16(4):563. doi: 10.3390/nu16040563 (PMC10892877; doi:10.3390/nu16040563)
Supplement: Supplementary file 1 [file nutrients-16-00563-s001.zip › nutrients-2878149-supplementary.pdf]

Supplemental Figure

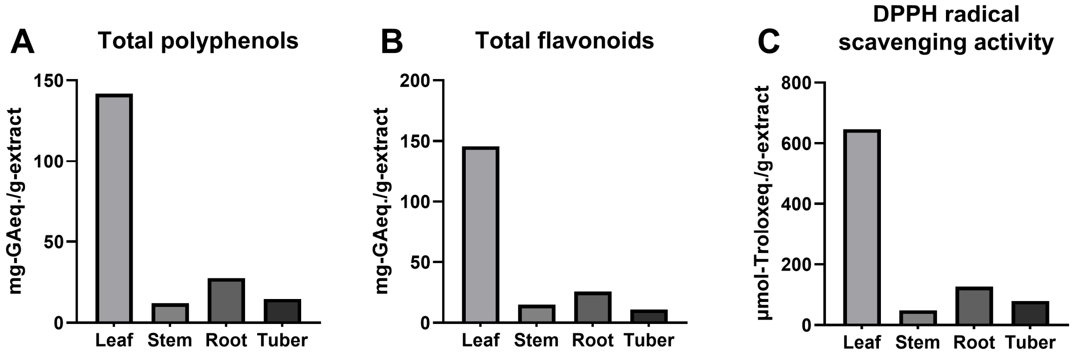

Figure S1. Total polyphenols, total flavonoids, and DPPH radical scavenging activity of sweet potato extracts

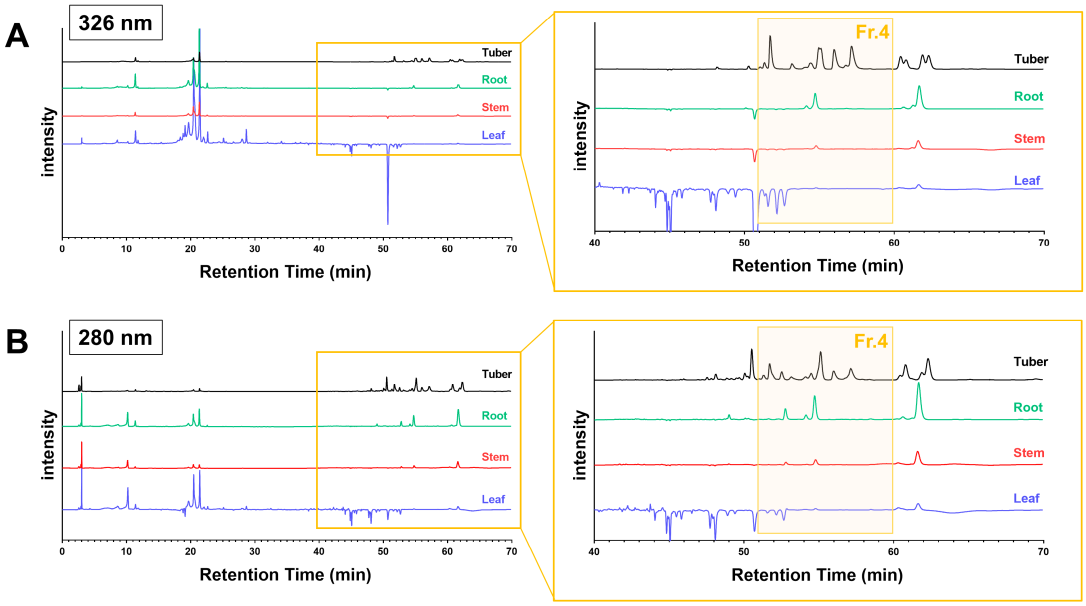

Figure S2. Comparison of peaks observed in DAD-HPLC analysis of sweet potato extracts
